# Consensus Standard for Evidence Integration into EMS Education and High-Stakes Testing

**DOI:** 10.1017/S1049023X2300047X

**Published:** 2023-06

**Authors:** Christopher B. Gage, Mark Terry, Kim D. McKenna, Jonathan R. Powell, Megan Hollern, Matt Ozanich, Christopher T. Richards, Christian Martin-Gill, Ashish R. Panchal

**Affiliations:** 1. National Registry of Emergency Medical Technicians, Columbus, Ohio USA; 2. The Ohio State University College of Public Health, Columbus, Ohio USA; 3. St. Charles County Ambulance District, St. Peters, Missouri USA; 4. The University of Cincinnati, Cincinnati, Ohio USA; 5. University of Pittsburgh, Pittsburgh, Pennsylvania USA; 6. The Ohio State University Wexner Medical Center, Columbus, Ohio USA

**Keywords:** education, EMS, integration, testing

## Abstract

**Background:**

Incorporating emerging knowledge into Emergency Medical Service (EMS) competency assessments is critical to reflect current evidence-based out-of-hospital care. However, a standardized approach is needed to incorporate new evidence into EMS competency assessments because of the rapid pace of knowledge generation.

**Objective:**

The objective was to develop a framework to evaluate and integrate new source material into EMS competency assessments.

**Methods:**

The National Registry of Emergency Medical Technicians (National Registry) and the Prehospital Guidelines Consortium (PGC) convened a panel of experts. A Delphi method, consisting of virtual meetings and electronic surveys, was used to develop a Table of Evidence matrix that defines sources of EMS evidence. In Round One, participants listed all potential sources of evidence available to inform EMS education. In Round Two, participants categorized these sources into: (a) levels of evidence quality; and (b) type of source material. In Round Three, the panel revised a proposed Table of Evidence. Finally, in Round Four, participants provided recommendations on how each source should be incorporated into competency assessments depending on type and quality. Descriptive statistics were calculated with qualitative analyses conducted by two independent reviewers and a third arbitrator.

**Results:**

In Round One, 24 sources of evidence were identified. In Round Two, these were classified into high- (n = 4), medium- (n = 15), and low-quality (n = 5) of evidence, followed by categorization by purpose into providing recommendations (n = 10), primary research (n = 7), and educational content (n = 7). In Round Three, the Table of Evidence was revised based on participant feedback. In Round Four, the panel developed a tiered system of evidence integration from immediate incorporation of high-quality sources to more stringent requirements for lower-quality sources.

**Conclusion:**

The Table of Evidence provides a framework for the rapid and standardized incorporation of new source material into EMS competency assessments. Future goals are to evaluate the application of the Table of Evidence framework in initial and continued competency assessments.

## Introduction

Emergency Medical Service (EMS) clinicians require current evidence-based, state-of-the-art education to optimize patient care provided in the prehospital setting,^1^ a concept encouraged by the EMS Agenda 2050.^2^ Evidence-based national guidance documents have been developed to support literature integration. Still, they are challenged due to the vast amount of scientific literature produced, the need to review the data, and its large variability in quality.^3^ To address this, the Prehospital Guidelines Consortium (PGC; United States) created a repository of evidence-based guidelines (EBGs) and a mechanism to evaluate the quality of these documents for potential integration into educational frameworks.^3–5^ Yet, significant gaps exist among available prehospital guidelines,^5^ which do not address all aspects of EMS medicine.^4,6^ Therefore, knowledge from primary scientific literature and other available educational materials must be integrated into prehospital care while identifying knowledge gaps for future evaluation.

Unfortunately, there are no rapid mechanisms to integrate new, high-quality evidence or guideline recommendations into the EMS educational and credentialing systems, including initial training, continued competency, and high-stakes assessment.^7^ The educational structure is framed by the national EMS education standards, core content, and scope of practice to set the standard and provide guidance for educational curricula.^2,8^ These educational systems, though rigorous, are updated infrequently and are not designed to address changes that require immediate implementation. For example, when rapid, impactful evidence was published in 2015 and 2016 concerning resuscitation and spinal motion guidelines, the National Registry of Emergency Medical Technicians (National Registry; Columbus, Ohio USA) released statements describing the emergent integration of these concepts into certification testing, driving immediate integration of this content at all levels of education. However, a precise mechanism was unavailable to facilitate integration, and the community leaned on position statements by the certification organization for evidence integration.^9^ This delay in evidence integration creates gaps between the recognized evidence-enhancing patient outcomes with education and prehospital services implementation. Another complication for evidence integration is the quality of the evidence being reviewed. Recognizing the vast heterogeneity in the available sources of materials for EMS clinicians (eg, blog posts, podcasts, systematic reviews, and guidelines), the data quality may only be apparent to some content consumers. In the absence of mechanisms to identify high-quality primary source material, EMS clinicians and EMS educators may need assistance identifying high-priority primary data sources to prioritize in EMS education that aligns with certification.

Therefore, a standardized method and hierarchy for integrating emerging evidence and knowledge into educational curricula or high-stakes examinations for EMS are necessary to optimize patient care. The objective was to obtain consensus from prehospital care experts to develop a valid, consistent, and transparent process for integrating and implementing new science into educational curricula and high-stakes testing, specifically how the National Registry will identify high- and low-quality emerging source material as a guide for EMS educators and clinicians when incorporating primary source material into educational content.

## Methods

### Study Design, Setting, and Participants

Accredited by the National Commission for Certifying Agencies (NCCA; Washington, DC USA), the National Registry is a nonprofit organization that provides certifications for EMS clinicians in more than 46 states, territories, and federal agencies.^10^ Using a modified Delphi evaluation to gain consensus from prehospital experts on implementing valid medical knowledge into the educational curriculum and the national certification examinations, the National Registry convened a Task Force (TF) in July 2020 that consisted of 11 subject matter experts in clinical practice, prehospital research, educational programming, EMS medical direction, and evidence review experts from the PGC (Appendix 1; available online only). The purpose of the TF was to provide recommendations to: (1) categorize medical evidence into a level of evidence hierarchy; and (2) implement these data into the national EMS certification examinations and education. The American Institutes of Research (Arlington, Virginia USA) IRB deemed this study exempted (Protocol number IRB00000436).

### Data Collection and Analysis

**Figure 1.**
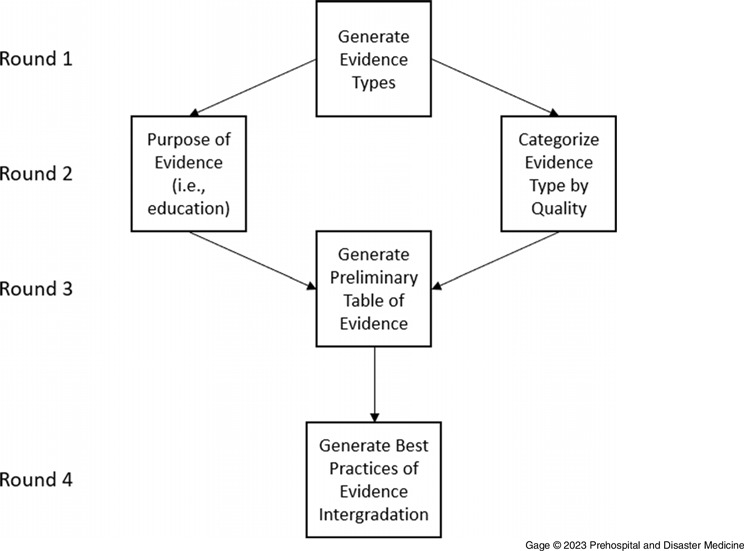
An Overview of the Work Performed in Each Round of the Modified Delphi Process.

A four-round modified Delphi method (Figure 1) was used to obtain group consensus.^11^ Rounds were done synchronously, and follow-up surveys were sent. When surveys were used, participation reminders were sent out one to two weeks after each round’s invitation, following Dillman’s tailored design methodology to improve survey response and participation.^12^ The primary outcome of interest was the development of consensus around the type and quality of prehospital evidence and the impact of integration into the national certification examination. Data were collected through online surveys utilizing the 2020 Alchemer, LLC (Lewisville, Colorado USA) survey platform.^13^

*Round One—*The initial round of data collection focused on having the experts identify potential source material available to EMS clinicians, EMS educators, and certification organizations. With many sources available to readers, understanding the specific types of evidence that may describe the standards of prehospital care is critical. Experts in Round One defined the potential sources and were asked to assign a categorical description of each type of evidence, including Primary Research, Recommendations for Care, and Information and Educational Content. In answering this query, participants were asked to consider a broad view of all the domains of EMS care, from cognitive to operational, and the associated literature that may inform decisions in these areas (eg, medical literature, National Institute for Occupational Safety and Health [NIOSH; Washington, DC USA] standards, and legal documents). Data were collected in a real-time interactive virtual meeting where all participants could brainstorm and generate ideas. Responses were collected and tabulated for the panel to evaluate.

*Round Two—*In Round Two, participants were asked to complete two tasks using the information from Round One. First, participants were asked to categorize each type of evidence identified in Round One into specific levels of evidence quality. As a baseline, participants were provided a framework of “high, medium, and low” levels of evidence that would describe the reliability and rigor of the evidence. Second, each participant also identified the evidence as either serving the purpose of “providing recommendations for diagnosis or care in the prehospital setting,” “primary research that does not provide care recommendations but reports data or evidence,” or “informational or educational content.”

*Round Three—*In Round Three, participants were asked to revise their judgments and provide feedback on the newly created Table of Evidence from data generated in the first two rounds. The table used a 3x3 format with rows being “Quality of Evidence” from high to low and columns being “Types of Evidence” as “Recommendations for Care,” “Primary Research,” and “Informational or Educational Content.” The TF then voted to categorize the evidence type with the appropriate quality level. The votes were tallied and shared with the members. This activity occurred in a virtual meeting with opportunities for each participant to provide feedback.

*Round Four—*During the final round, the recommendations from the previous rounds were discussed, and feedback was requested concerning the generated Table of Evidence. The TF was then asked to define the best practices for integrating each evidence level into certification examination frameworks, including time goals. Finally, to support the discussion on the impact of evidence integration on certification examination processes, the examinations team from the National Registry provided additional details on exam development processes and production cadences.

### Data Analysis

The primary outcome was the development of a unanimous consensus around the type and quality of prehospital evidence and the impact on integration into the national certification examination. Descriptive statistics were calculated for each round using Stata IC 17 (StataCorp LP; College Station, Texas USA).^14^ For Round Two, quality of evidence data for each evidence type were collected, and high-, medium-, and low-quality responses were coded with scores of six, three, and one. Average scores for evidence quality were then tabulated and provided to the TF. These scores were used as a guide for final categorization into high-, medium-, and low-quality evidence categories.

## Results

The TF members gathered virtually in July 2020, where they were introduced to the overall project’s goals and the study plan. In Round One, the TF members (Appendix 1) generated a list of potential content sources that could be interpreted as informing EMS education or certifying exam content. Appendix 2 (available online only) shows these sources with a definition or example, recognizing that any particular content source may fit multiple types. In addition, the panel also identified potential categories that these sources of content could be subdivided into, including evidence that provided “recommendations for patient care” or sources of “primary research.” But again, these were examples of distinct categories, not a comprehensive list.

In Round Two, 17 evidence types aligned with “recommendations of care” and seven with “primary research.” The TF was also asked to evaluate the quality of each content source (Table 1). Each source was categorized into high-, medium-, and low-quality. Only four types were placed in the “high quality of evidence” category, including EBGs (meeting National Academy of Medicine [NAM; Washington, DC USA] criteria), meta-analysis studies, randomized controlled studies, and systematic reviews. The NAM criteria describe concrete recommendations that define high-quality, EBGs and are provided in Appendix 3 (available online only).^4,6,7,13,15–17^ All other sources were categorized as medium or low levels of evidence. Since the medium-quality of evidence category was noted to have significant internal heterogeneity in the quality of evidence, the panel recommended a subdivision and ranking (I, II, and III) within this category to allow for more accurate stratification (Table 1).

**Table 1.** Distribution of Types of Evidence by the Quality Rating as Defined by the Task Force

High Quality of Evidence	Medium Quality of Evidence		Low Quality of Evidence
Evidence-Based Guideline (meeting NAM criteria)	I	Government Standards	Blogs/Podcasts
Meta-Analysis		Legal Briefs/Court Opinion	Case Series or Reports
Randomized Controlled Study (blinded or other)		Regulatory Standards	Expert Lecture
Systematic Review	II	Education Standards	Expert Opinion
		Evidence-Based Guidelines (not meeting NAM criteria)	Informal Crowd Source Projects
		National Model Clinical Guidelines	
		Observational Study	
		Position Statement	
		Retrospective Analysis	
		Structured Training Courses	
		Quasi-Randomized Trials	
	III	Best Practice Documents	
		Technical Reports	
		Textbooks	

Note: Each source of evidence is listed in alphabetical order within each Quality of Evidence category. Medium quality of evidence was subdivided and ranked (I, II, III).

Abbreviation: NAM, National Academy of Medicine.

**Figure 2.**
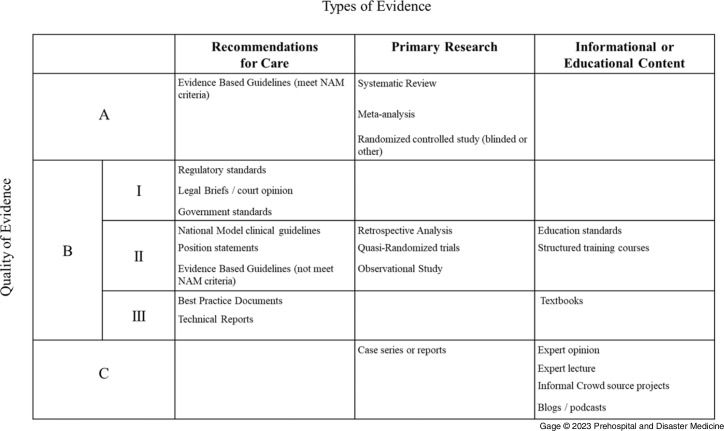
Final Combined Table of Evidence Developed by the Task Force Panel. Abbreviation: NAM, National Academy of Medicine.

In Round Three, the TF provided feedback on the generated tables from Round Two. After evaluating the initial iteration of the categories with which the evidence types aligned, the TF identified the need to add another category of evidence type entitled “Informational and Educational Content” (Table 2). This decision was made to capture content created explicitly as education for EMS clinicians, allowing a more efficient placement into the evidence hierarchy. The panel also conducted small iterative changes of the position in the table of some types of evidence. No fundamental changes in the overall structure were made. The final categories for all evidence types were updated, and a unanimous consensus was reached defining the final table of evidence (Figure 2).

**Table 2.** Distribution of Types of Evidence into Categories Defined by the Task Force

Recommendations for Care	Primary Research	Information and Educational Content
Best Practice Documents	Case Series or Reports	Blogs or Podcasts
Evidence-Based Guidelines (meeting NAM criteria)	Meta-Analyses	Education Standards
Evidence-Based Guidelines (not meeting NAM criteria)	Observational Studies	Expert Lectures
Government Standards	Quasi-Randomized Trials	Expert Opinions
Legal Briefs/Court Opinions	Randomized Controlled Studies (blinded or other)	Informal Crowd Source Projects
National Model Clinical Guidelines	Retrospective Analyses	Structured Training Courses
Position Statements	Systematic Reviews	Textbooks
Regulatory Standards		
Technical Reports		

Abbreviation: NAM, National Academy of Medicine.

**Figure 3.**
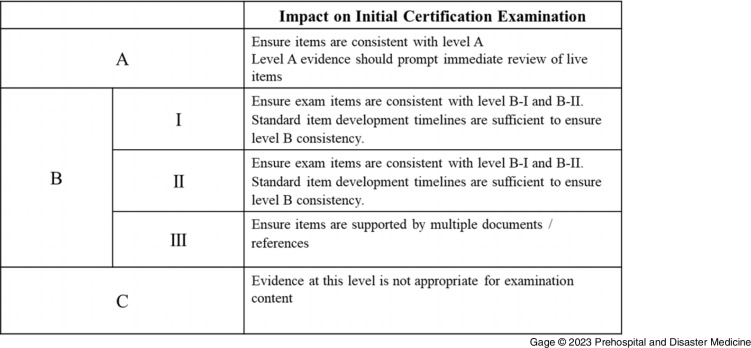
Task Force Recommendations Concerning the Impact of Evidence on Certification Examination Based on the Quality of Evidence.

Finally, in Round Four, the TF was tasked to provide recommendations, using the Table of Evidence, for best practices for integrating evidence into the National Registry certification examination (Figure 3). The TF noted concerns about integrating low-level evidence into assessment structures because low levels of evidence may be error-prone, inconsistent, and not rigorous enough to develop reliable examination items. Thus, the TF recommended that examinations not reference content from publications designated as low-quality evidence. Due to similar concerns, the TF recommended that any single B-III evidence source should not be the sole reference for examination items. Items drawing from content in B-III sources need additional sources. In comparison, high-quality evidence (A) could suffice as a standalone reference.

The TF also commented on the timing of the necessary changes to examination content when new evidence is created. First, evidence that satisfies the A level of evidence should immediately impact the certification examination. Therefore, examination items currently in use that are affected by this evidence should be immediately reviewed for content. Concerning level B evidence sources, the panel recommended that any examination items be consistent with this evidence. However, the standard item development timelines for updates are sufficient for item revision when new evidence is published at this level.

The TF considered the implications of the timetable for adapting examinations to be consistent with new content. While eliminating items that conflict with new evidence can be accomplished quickly, subject matter experts with expertise in EMS educational systems estimated that developing and reviewing the latest content based on new content can take at least 12 months. Therefore, the TF did not formally recommend a specific timeline for content modification. However, after reviewing factors around the issue, a consensus among the group supports a regular biennial process. A biennial process allows consolidated reviews, scheduled and anticipated communication of identified EBGs, integration with content development and publication cycles, and modification of educational content and procedures.

The TF further discussed the potential effects on the certification examination process, including examination publication schedules, depth and quantity of references, discrepancies between authorities, operational live item impacts, pre-test items that are correct but fail psychometric analysis due to lack of EBG dissemination, onboarding of examinations’ team members and subject matter experts, and leadership needs for new EBG implementation. The TF recommended that further clarity with policies and procedures to manage these potential impacts be developed to assist in the certification process. Furthermore, as new evidence sources are identified, the National Registry must create policies and procedures to implement these potential biennial reviews into examination content. For example, the PGC has previously completed a systematic review of EBGs and graded the quality of those guidelines using the Appraisal of Guidelines for Research & Evaluation (AGREE) II tool and a categorization scheme based on whether guidelines meet the NAM criteria for “Clinical Practice Guidelines We Can Trust.”^15^ If updated biennially, this systematic review could be used to revise examination content incorporating the latest scientific evidence and recommendations generated from these published guidelines.

## Discussion

This manuscript describes how prehospital care experts developed a robust and transparent process for integrating and implementing new evidence into educational curricula and high-stakes testing. Leveraging a modified Delphi process, experts developed a Table of Evidence that weighs the quality and type of evidence to assist educators and test designers with new evidence incorporation for initial certification and continuing education requirements. Additionally, this framework provides a process for immediate integration of high-quality content into EMS clinician education, both initial and through continuing education, to facilitate the implementation of high-quality content that can directly improve patient clinical outcomes.

This work is the first example of a systematic structure to guide new evidence integration into prehospital education and testing. Prior work has demonstrated that inconsistencies in evidence integration into clinical practice can lead to inappropriate or unsafe care.^18,19^ To improve safety, quality of care, and patient-centered outcomes, the prehospital educational system leverages certification as an independent validation to confirm provider knowledge, skills, and abilities.^20–22^ Though structures have been described for integrating evidence into continuing professional development, one continued theme is that timely information transfer from the release of new evidence to integration into teaching at the initial certification level is a significant barrier.^19^ Educators often depend on textbook manufacturers for content since this is one of the primary training tools used in the classroom setting.^23,24^ Still, with the current pace of new medical knowledge generation (on average, academic output doubled every 73 days in 2020^16^), the challenge for timely evidence integration into initial education is great. The structured paradigm for identifying high-quality evidence outlined in this manuscript can improve the process.

The developed Table of Evidence also addresses another challenge noted by the TF: variability in the quality of evidence available to EMS clinicians. As listed in Table 1, the TF identified high-quality primary source material with validated methodologies, such as EBGs, systemic reviews, and controlled trials. However, other sources were unvalidated and non-peer-reviewed such as expert opinions, informational crowdsourcing projects, or blogs/podcasts. Depending on the evidence and reliability of the information, some primary source material may need to be evaluated closely before dissemination. The process provides a framework for how EMS educators can address primary source material based on how the National Registry will view types of sources when creating examination items.

One critical next step the TF noted is to begin categorizing evidence applicable to prehospital care using the Table of Evidence. For prehospital EBGs, the PGC has curated a repository of EBGs and has reviewed the quality of evidence evaluation and development of recommendations for prehospital care within these guidelines.^4–6,25^ The PGC has also defined gaps in the content of prehospital guidelines that could improve patient outcomes.^4^ Applying structured approaches to primary source material can help identify high-quality evidence and prioritize implementing new knowledge by applying the Table of Evidence.

## Limitations

The analysis has several limitations, with many associated with the Delphi method. First, due to COVID-19, much of the TF’s work was done using electronic mail, online meetings, and survey platforms. However, the researchers leveraged serial debriefings in each round to manage possible limitations in electronic communications. Additionally, the consensus decisions reached by this group of subject matter experts result from their backgrounds and experiences in EMS. Therefore, it is possible that a different group of experts may have generated a different outcome for the Table of Evidence. Lastly, validation of the Table of Evidence framework for facilitating evidence integration into educational program curricula and continued competency platforms is an important future initiative.

## Conclusion

A Task Force of prehospital experts developed a Table of Evidence that will provide national, state, and local agencies with a more transparent mechanism for integrating new evidence into certification examinations and educational guidelines. Additionally, this framework places value on the immediate integration of high-quality content and a process for timely updates to educational curricula.

## Supplementary material

For supplementary material accompanying this paper visit https://doi.org/10.1017/S1049023X2300047X. click here to view supplementary material
