# Supplementary material for: Consensus Standard for Evidence Integration into EMS Education and High-Stakes Testing
Source: Prehosp Disaster Med. 2023 May 4;38(3):338–44. doi: 10.1017/S1049023X2300047X (PMC10267721; doi:10.1017/S1049023X2300047X)
Supplement: Supplementary file 1 [file S1049023X2300047Xsup001.docx]

**Appendix 1.** Task Force Members and their Associated Titles

| **Expert** | **Position Title** |
| --- | --- |
| Art Hsieh | Education Director and Chief Operating Officer, San Francisco Paramedic Association |
| Baxter Larmon | Director Emeritus of the UCLA Center for Prehospital Care |
| Deb Akers | Division Director for Accreditation, Certification, and Education, Virginia |
| Jose Cabanas | Chief Medical Officer for Wake County Government, NC |
| Paul Rosenberger | EMS Faculty at The University of Texas Southwestern Medical Center in Dallas & EMS Faculty at Tarrant County College |
| Juan March | Chief, Division of EMS, East Carolina University, NC |
| Walt Stoy | Director of the Office of Education at the Center for Emergency Medicine, University of Pittsburgh |
| Christian Martin-Gill | Chief, Division of EMS at the University of Pittsburgh Medical Center, President of Prehospital Guidelines Consortium |
| Christopher Richards | Associate Professor, Division of EMS, Department of Emergency Medicine, University of Cincinnati |
| Kathleen Brown | Medical Director of the Emergency Medicine and Trauma Center at Children’s National Hospital |
| Kim McKenna | Emeritus faculty of St. Charles County Ambulance District, NAEMSE National Faculty, National Registry of EMTs Board of Directors |

**Appendix 2.** Types of Evidence Representing Sources of Evidence for an EMS Certification Examination. These types of evidence are alphabetized and do not reflect priority.

| **Type of Evidence** | **Definition** |
| --- | --- |
| Best-Practice Documents | Eg, Centers for Disease Control and Prevention (CDC) Guidance, EMS model protocols (state or regional), and state guidance documents. |
| Blogs or Podcasts | Regularly updated website or web page (blog) or digital audio file (podcast) that is written in an informal or conversational style made available on the internet, Free Open Access Medical Education (FOAMed)). |
| Case Series or Reports | Published case series or reports. |
| Education Standards | Standards used for education curriculum development (eg, National EMS Education Standards). |
| Evidence-Based Guideline (Meeting NAM criteria) | Guidelines based on a systematic literature review using methods to assess evidence and review quality (eg, Grading of Recommendations, Assessment, Development and Evaluations (GRADE)) and provide concrete recommendations based on the evidence review. These guidelines will satisfy the framework set by the National Academy of Medicine (NAM) for Clinical Practice Guidelines We Can Trust. |
| Evidence-Based Guideline (Not meeting NAM criteria) | Guidelines that provide recommendations based on an organized literature review but do not meet all the requirements outlined by the National Academy of Medicine for Clinical Practice Guidelines We Can Trust. They may include scoping or directed scientific literature reviews or be based primarily on expert or consensus-based recommendations. |
| Expert Lecture | Lectures provided by noted experts in a specific topic area. |
| Expert Opinion | Written expert opinion documents (including online). |
| Government Standard | Eg, American Society for Testing and Materials (ASTM) <https://www.astm.org/COMMIT/SUBCOMMIT/F30.htm>. |
| Informal Crowd Source Project | Obtaining work, information, or opinions from a large group of people who submit their data via the Internet, social media, and smartphone apps (eg, Wikipedia). |
| Legal Briefs/Court Opinion | A statement that is prepared by a judge or court announcing the decision after a case is tried, establishing standards. |
| Meta-Analysis | Examination of data from several independent studies of the same subject to determine overall trends. |
| National Model EMS Clinical Guidelines | Guidelines based on a combination of evidence evaluation and expert consensus, supported by the National Association of State EMS Officials (NASEMSO) and the National Highway Traffic Safety Administration (NHTSA) (https://nasemso.org/wp-content/uploads/National-Model-EMS-Clinical-Guidelines_2022.pdf). |
| Observational Study | Correlational (ie, non-experimental) research in which a researcher observes ongoing behavior. |
| Organizational Website | Informational website supported by public/private organizations (eg, mayoclinic.org). |
| Peer Review by Informal Process | Review of completed research work by informal peer review process (eg, Cureus Journal of Medical Sciences). |
| Position Statement | Organizational position statement or joint statement, often based on a combination of evidence evaluation and expert consensus (eg, “EMS Spinal Precautions and the Use of the Long Backboard,” “Clinical Care and Restraint of Agitated or Combative Patients by Emergency Medical Services Practitioners”). |
| Quasi-Randomized Trial | A study where participants are allocated to different arms of the trial (eg, to receive the study medicine or placebo) using a method of allocation that is not truly random. |
| Randomized-Controlled Trial  (Blinded or other) | A study design that randomly assigns participants into an experimental group or a control group. |
| Regulatory Standard | Eg, National Transportation Safety Board standards, National Fire Protection Association standards |
| Retrospective Analysis | A study that uses existing data recorded for reasons other than research. |
| Structured Training Courses | Courses developed and delivered by recognized organizations with clear objectives and certifications following completion (eg, Advanced Cardiac Life Support (ACLS), Advanced Stroke Life Support (ASLS), Emergency Neurological Life Support (ENLS), International Trauma Life Support (ITLS), Prehospital Trauma Life Support (PHTLS)). |
| Systematic Review | Eg, Cochrane Network Systematic Reviews and International Liaison Committees on Resuscitation (ILCOR) reviews. |
| Technical Reports | Written documents detailing the results of a project and submitted to the sponsor of that project that are not peer-reviewed unless they are subsequently published in a journal. |
| Textbooks | Eg, Prehospital Emergency Care, Emergency Care, Sanders’ Paramedic Textbook, Paramedic Care: Principles & Practice |
| Trade Journals | Periodicals containing news and items of interest concerning a particular trade. |

**Appendix 3.** National Academy of Medicine Inclusion Criteria for Clinical Practice Guidelines. Adapted from the National Guideline Clearinghouse,^25^ the National Academy of Medicine,^15^ and the Prehospital Guidelines Consortium.^4,6^

| **Criteria** | **Description** |
| --- | --- |
| 1. Systematically Developed Recommendations | The clinical practice guideline contains systematically developed statements, including recommendations intended to optimize patient care and assist physicians and/or other health care practitioners and patients in making decisions about appropriate health care for specific clinical circumstances. |
| 1. By an Association or Similar Organization | The clinical practice guideline was produced under the auspices of a medical specialty association; relevant professional society; public or private organization; government agency at the Federal, State, or local level; or health care organization or plan. A clinical practice guideline developed and issued by an individual(s) not officially sponsored or supported by one of the above types of organizations does not meet the criteria. |
| 1. Systematic Review | The clinical practice guideline is based on a systematic review of the evidence as demonstrated by documentation of each of the following features in the clinical practice guideline or its supporting documents. |
| - 1. Statement | An explicit statement that the clinical practice guideline was based on a systematic review.* |
| - 1. Search Strategy | A description of the search strategy that includes: - A listing of database(s) searched, - A summary of search terms used, and - The specific period covered by the literature search, including the beginning date (month/year) and end date (month/year) |
| - 1. Study Selection | A description of the study selection that includes: - The number of studies identified, - The number of studies included, and - A summary of inclusion and exclusion criteria. |
| - 1. Synthesis of Evidence | A synthesis of evidence from the selected studies, eg, a detailed description or evidence tables. |
| - 1. Summary of Evidence Synthesis | A summary of the evidence synthesis (see 3d above) included in the guideline that relates the evidence to the recommendations, eg, a descriptive summary or summary tables. |
| 1. Assessment of Benefits/Harms and Alternative Care Options | The clinical practice guideline or supporting documents assessing the benefits and harms of recommended and alternative care options. |
| 1. English and to the Public | The complete guideline is available in English to the public upon request (for free or for a fee). |
| 1. Current | The guideline is current and the most recent version.** |

* If an explicit statement that the clinical practice guideline was based on a systematic review was not provided, but all other criteria and subcriteria describing a systematic review were determined to be present, that subcriterion was marked as complete. Guideline developers should be advised to contain such an explicit statement in future guidelines.

** The NGC Inclusion Criteria and NAM (formerly the Institute of Medicine) recommends that a guideline be developed, reviewed, or revised within the past five years. For this systematic review, this criterion was waived.
